# Evaluating the impact of segmentation strategies on radiomic feature stability for paediatric brain tumour diagnosis using diffusion weighted imaging

**DOI:** 10.1371/journal.pone.0356382

**Published:** 2026-08-18

**Authors:** Timothy Mulvany, Daniel Griffiths-King, Lara Worthington, Katherine Crombie, Heather E. L. Rose, Andrew Peet, John Apps, Jan Novak

**Affiliations:** 1 Aston Institute of Health and Neurodevelopment, Aston University, Birmingham, United Kingdom; 2 Department of Oncology, Birmingham Women’s and Children’s Hospital NHS Foundation Trust, Birmingham, United Kingdom; 3 Institute of Cancer & Genomics Science, University of Birmingham, Birmingham, United Kingdom; 4 Medical Physics Nottingham University Hospitals NHS Trust, Nottingham, United Kingdom; 5 School of Medicine, University of Nottingham, Nottingham, United Kingdom; University of Pisa, ITALY

## Abstract

**Objectives:**

This research aims to comprehensively assess the impact of both conservatively and extensively delineated Regions of Interest (ROI) on subsequent quantitative in-vivo characterisation of paediatric brain tumours using diffusion-weighted MRI.

**Methods:**

Utilising a retrospective cohort of 106 paediatric brain tumour patients, ground truth (GT) ROIs delineating tumour boundaries were eroded or dilated, simulating conservative and extensive ROI drawing strategies respectively. Stability was evaluated for 19 first-order radiomic features extracted from Apparent Diffusion Coefficient (ADC) maps within each ROI. Further, these features were used to train a series of machine learning models to evaluate the impact of ROI boundaries on downstream diagnostic classification.

**Results:**

For 18/19 first-order features, ROI dilation introduced significantly (*p* < 0.01) greater feature variability than erosion, with large effect size (d > 0.8) for 11 features. This relationship was variable between diagnoses, and strongest amongst pilocytic astrocytomas. Diagnostic models trained using features from GT ROIs were negatively impacted with classification accuracy reduced by 3.8 ± 0.8% and 5.6 ± 0.9% for low-level erosion and dilation respectively. Inclusion of eroded/dilated ROI features into the training dataset combined with stable-feature selection partially mitigated the impact of erosion/dilation on model accuracy with 1.4 ± 0.7% and 2.9 ± 0.3% accuracy drop compared model accuracy on features extracted from GT ROIs.

**Conclusion:**

The consistently reduced impact of conservative boundaries over extensive ones suggests that, in terms of segmentation strategies, exclusion of ambiguous boundary regions may be preferable over their inclusion. Additionally, diagnostic models exhibited improved robustness to variable ROI drawing strategies through training augmentation and selection of stable features.

## 1 Introduction

Rapid and accurate diagnosis, alongside effective risk stratification, is critical for determining optimal treatment pathways for paediatric brain tumours, to maximise patient outcomes. Leveraging MRI acquired prior to surgical intervention, for the purposes of early, in-vivo characterisation of brain tumours remains a key goal of neuro-radiological research.

Brain tumour research often leverages diffusion-weighted MRI imaging (DWI), specifically through analysis of apparent diffusion coefficient (ADC). Quantifying the pointwise magnitude of water molecule diffusion within tissues, spatial maps of ADC offer valuable insights into tumour cellularity [1].

Radiomics, the practice of extracting quantitative features from image subregions, enables pathology characterisation in a repeatable and standardised way [2]. Such quantitative methodologies are well-suited to the field of medical image analysis, transforming high-dimensional imaging containing complex pathology of varying shapes and sizes, to a smaller standard set of objective and comparable data points, making radiomics a promising tool for improved in-vivo tumour characterisation and treatment decision-making [3]. Previous research has demonstrated that quantitative, radiomic features extracted from ADC maps can provide accurate diagnostic classification of paediatric brain tumours [4–8].

However, DWI exhibits low signal-to-noise ratio and is highly sensitive to magnetic-field heterogeneity [9], leading to increased variability in Region of Interest (ROI) drawing, both between annotators and individually [10; 11]. This is further compounded in paediatrics by ongoing brain development, patient movement causing motion artefacts [12], lower occurrence of brain tumours in children than in adults [13], and absence of well-defined segmentation guidelines. Variations in segmented tumour boundaries will alter any extracted radiomic feature values, potentially impacting their utility in radiological tools.

Research typically employs highly accurate ROIs to prototype radiological tools, drawn through lengthy collaborative/iterative workflows, and are likely more robust than those drawn under clinical time constraints by an individual annotator. To enable the clinical application of quantitative features extracted from ADC, it is crucial to evaluate their susceptibility to typical annotation biases, specifically over- and under-segmentation, and the impact on downstream clinical tasks such as automatic diagnosis.

Previous studies have investigated diagnostically homogenous cohorts, utilising segmentation perturbations for feature selection, identifying stable features within their specific domains [14–16]. Instead, this study investigates the estimates the quantitative impact of paediatric brain tumour segmentation biases on radiomic feature stability and subsequent downstream model performance, employing in-silico simulation of conservative and extensive ROIs. This research aims to provide guidance for improving the robustness and generalisability of radiomic models for paediatric brain tumour analysis.

## 2 Materials and methods

### 2.1 Data

#### 2.1.1 Cohort.

This study utilised retrospective MRI and clinical data of paediatric brain tumour patients recruited into the Imaging of Tumours (IoT) study (CNS 2004 10) with informed consent provided by parents or guardians. Approved by NHS research ethics committee (04/MRE04/41), imaging was performed at Birmingham Children’s Hospital between 25/07/2005 − 04/01/2022 as part of standard of care prior to any surgical intervention or treatment, with retrospective IoT Study recruitment between 08/08/2006–29/12/2023. Data handling and methods used in this study were performed in accordance with all relevant regulations.

**Fig 1:**
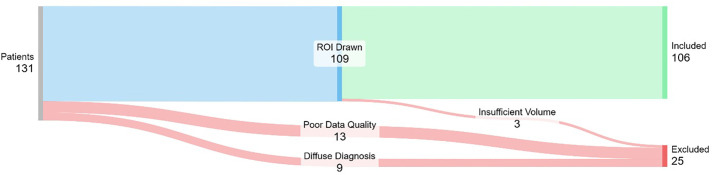
Patient exclusion workflow, on basis of poor image quality, diffuse diagnoses and insufficient tumour volume. Of 131 patients, 109 were successfully annotated, and 106 were included for use in this study.

A cohort of 131 participants from the original study were initially identified, requiring that participants were aged 0–18 with pre-surgical DWI available on the study’s database. A subset of 22 participants were subsequently excluded prior to ROI drawing as shown in Fig 1, due to:

- (N = 13) Presence of artefacts that visibly/significantly disrupted DWI where there was pathology (determined by TM, JN and DGK)
- (N = 9) Diffuse tumours (e.g., Diffuse Midline Glioma) where accurate tumour margins were determined to be unclear.

Three participants were excluded following ROI drawing due to insufficient tumour volume (required ≥100 voxels in ≥2 axial slices).

The resulting cohort of N = 106 participants included 25 distinct diagnostic classifications (detailed in Supplementary Material). The three largest diagnostic groups were(N = 30) Pilocytic Astrocytomas (PAs), (N = 27) Medulloblastomas (MBs) (including Anaplastic and Desmoplastic-Nodular), and (N = 12) Ependymomas (EPs) (including Anaplastic) and are among the most prevalent in clinical presentation [17]. Diagnoses were ascertained according to WHO histological diagnosis where available, and radiological diagnosis where biopsy was not performed.

Age and gender distributions are reported in Table 1. A diagnostic subset (N = 69) containing only PAs, MBs, and EPs was used for analysing downstream diagnostic task performance. Differences in distributions of age and gender were small and non-significant.

**Table 1:** Cohort Demographic Details.

	Full Cohort	Diagnostic Subset	Significance
Number of Patients	106	69	–
Gender split (F:M)	53:53	37:32	*p* = 0.32
Mean (SD) Age (Years)	6.9 (4.6)	7.0 (4.5)	*p* = 0.84
Age Range (Years)	0.0-16.3	0.3-16.3	–

*Age = Age at diagnosis, SD = Standard Deviation, F:M = Female:Male, significance of the subset’s gender split is calculated from one-tailed binomial test n = 69, p = 0.5, k = 32, and significance of change in age distributions calculated from one-tailed Mann-Whitney U test.

#### 2.1.2 Image acquisition.

Pre-surgical DWI was acquired at 1.5T or 3T for each participant across multiple MRI scanners, (Table 2), all using diffusion b-values of 0 & 1000 s/mm^2^ (b0 & b1000). ADC maps were calculated using standard voxel-wise equation:

**Table 2:** MRI Scanner Details and Image Dimensions.

Scanner	Scanner	Field	Number of	In-plane	Slice
Manufacturer	Model	Strength (T)	Patients (N)	Resolution (mm)	Thickness (mm)
Siemens	Avanto	1.5	55	1.2	4.0 - 6.5
GE HealthCare	HDxt	1.5	20	0.9	5.0 - 6.0
Philips	Achieva	3	13	0.9	5.0 - 6.0
Siemens	Symphony	1.5	12	1.6 - 1.8	5.2 - 6.5
Siemens	Aera	1.5	6	0.6	4.4 - 5.2

*Each patient scanned on one of above scanners.


$$\begin{array}{c} \text{ADC}=\frac{-\text{ln}\left(S_{b}/S_{\text{0}}\right)}{b} \end{array}$$

where $S_{b}$ is the observed signal intensity with diffusion weighting of b, and $S_{\text{0}}$ is the observed b0 signal. ADC values were truncated between 0−4 × 10^-3^mm^2^/s, as is common practice [14; 15]. No further image corrections were performed, as ADC maps have demonstrated good reproducibility regardless of acquisition parameters [18].

#### 2.1.3 ROI drawing.

ROIs were manually annotated as overlays on b0 images, using b1000 as main reference, and (pre- and post-contrast) T1-weighted, and T2-weighted MRI as additional references for especially difficult or ambiguous tumour sub-regions with insufficient DWI contrast. ROIs included solid tumour only, excluding oedema and cystic regions.

Internal regions defined as cystic, required a minimum of 4 contiguous pixels within each axial slice, mitigating against exclusion of small, non-cystic regions resulting from noise and artefacts present in lower-quality clinical imaging. Tumours had to be visible across multiple axial slices, with ≥100 voxels in ≥2 slices, ensuring sufficient volume to endure erosion methodologies, resulting in 3 participants being excluded following ROI drawing (see Fig 1).

ROIs were collaboratively developed and iteratively refined. TM annotated and revised ROIs, with repeated reviewed by expert scientists JN & DGK, with 13- and 9-years’ experience respectively, spanning neuroimaging and paediatric MRI research. 3D Slicer [19] (version 5.2.1) was used for ROI drawing.

#### 2.1.4 ROI manipulation.

**Fig 2:**
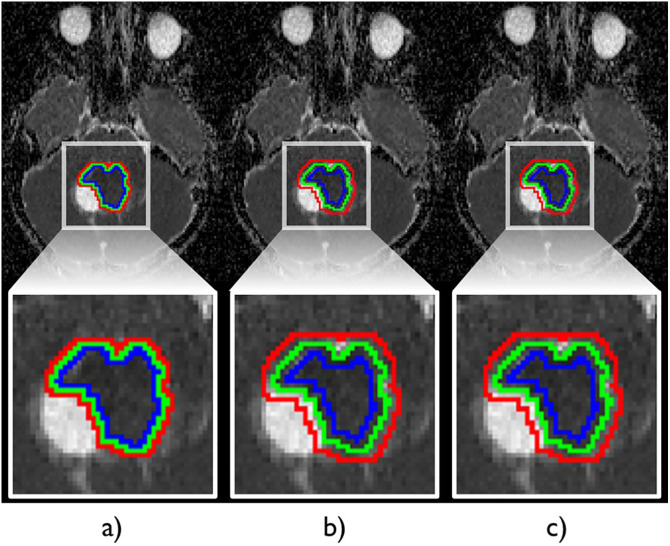
Example of manually delineated boundary of a medulloblastoma and resulting boundary changes following increasing levels of erosion and dilation. With the GT boundary in green, the dilated boundary in red and the eroded boundary in blue, images display a) 1 iteration b) 2 iterations c) 3 iterations of erosion and dilation. ROIs are inclusive of marked boundary.

ROI boundaries were adjusted through 2D-convolutional erosion and dilation of each axial slice, imitating overly conservative or extensive drawing strategies respectively. Operations were performed using OpenCV [20] (version 4.8.1), utilising 3x3 all-ones kernels and applied up to 3 times consecutively to assess increasing degrees of variability, as demonstrated by an example in Fig 2.

#### 2.1.5 Feature extraction.

All 19 first-order features defined by PyRadiomics [21] v3.1.0 were extracted from the subregions of each participant’s ADC map, defined by ground-truth, eroded, and dilated 3D ROI boundaries. A bin width of 25 × 10^−6^ mm^2^/s was utilised for calculating histogram-based metrics, such as entropy and uniformity. Features were calculated following isotropic (1 mm × 1 mm × 1 mm) resampling of the original ADC volume using PyRadiomics’ built-in methods, applying nearest neighbour and linear interpolation for ROIs and ADC volumes respectively.

Due to the highly variable acquisition parameters, and the large slice gaps present in DWI/ADC images, as seen in Table 2: MRI Scanner Details and Image Dimensions Table 2, the textural features provided by PyRadiomics were not evaluated in this research. Furthermore, the literature surrounding diagnostic classification of paediatric brain tumours conventionally focuses on first-order quantitative features only due to their interpretability and availability in standard clinical reporting tools.

The resulting 7 feature sets containing 19 features for each of the included 106 participants are hereafter referred to as:

- E × 1, E × 2, E × 3: feature values extracted using ROIs following 1, 2, and 3 iterations of boundary erosion (respectively)
- D × 1, D × 2, D × 3: feature values extracted using ROIs following 1, 2, and 3 iterations of boundary dilation
- GT – feature values extracted using ground-truth ROIs

GT feature values were normalised across the cohort to enable direct comparison of features with vastly different scales. E × 1–3 and D × 1–3 were transformed using GT normalisation parameters.

### 2.2 Analytical methods

#### 2.2.1 Exp. 1: Feature stability.

The impact of erosion and dilation on the resulting quantitative feature values was assessed via measuring the average absolute difference relative to the GT at the patient level. The mean “impact” was also calculated, for the full cohort, alongside PAs, MBs, and EPs individually, evaluating the relative susceptibility of each diagnosis to ROI variations.

Two-tailed, paired t-tests (for each of the 19 features) assessed significance of absolute differences (relative to GT features) caused by erosion vs. dilation (E × 1, D × 1), with effect size measured by Cohen’s d [22]. A Bonferroni corrected significance level was utilised (α_corr._ = 0.0026).

Average histogram profiles of ADC were plotted for each common diagnosis (EPs, MBs, and PAs) for each level of erosion and dilation. Histograms were volume-normalised prior to averaging to prevent volume bias.

#### 2.2.2 Diagnostic models for Exp. 2–4.

To assess the impact of ROI variability on diagnostic prediction a set of Random Forest (RF) classifiers were trained to differentiate between PAs, MBs, and EPs, implemented via Python (v3.10) using scikit-learn (v1.1.3). Additional diagnoses possessed insufficient quantity for training stable models.

RFs utilised default hyperparameters except for maximum tree depth of 3 identified through exhaustive search, reducing overfitting by limiting tree complexity. Class weightings mitigated against diagnostic class imbalance. All models were trained and evaluated using patient-wise, leave-one-out cross validation (LOOCV). When evaluating on a given participant, training data excluded any/all ROIs from that patient, GT and adjusted. Performance was assessed through overall model accuracy. Experiments were repeated 100 times to acquire 95% confidence bounds due to stochastic behaviour of RFs.

#### 2.2.3 Exp. 2: Optimal single ROI drawing strategy.

**Fig 3:**
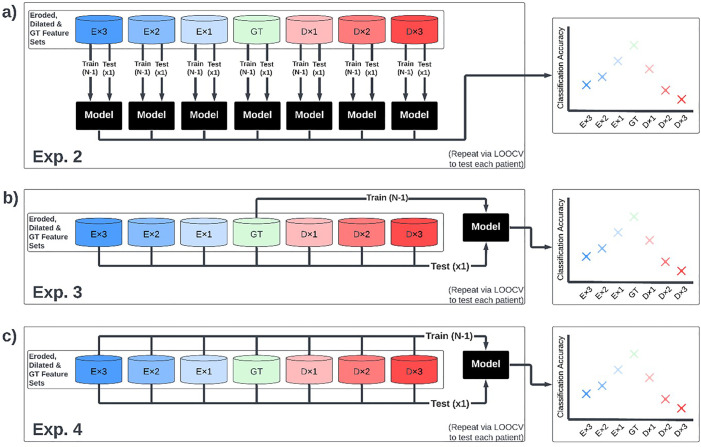
Flowcharts depicting model training and evaluation workflows for a) Exp. 2, b) Exp. 3, c) Exp. 4. Models are trained via LOOCV with each loop trained using relevant feature sets from 68 patients (N-1) and evaluated on the 1 remaining patient’s corresponding feature sets. Graphs are not representative of results.

To establish whether conventional ground-truth ROIs capturing precise tumour boundaries provide optimal diagnostic performance, 7 sets of diagnostic models were trained and evaluated each using one of the 7 available feature sets (see Fig 3).

#### 2.2.4 Exp. 3: Impact on existing trained diagnostic models.

This experiment sought to establish the robustness of models, trained on GT ROIs, when subsequently applied to ROIs with conservative or extensive biases. A set of models were trained utilising only GT features values, and subsequently evaluated on features extracted from each level of erosion and dilation (see Fig 3).

#### 2.2.5 Exp. 4: Mitigating impact and improving model robustness.

Finally, this study investigated potential methods for mitigating any observed impact to model performance arising from annotator biases, improving model robustness.

Firstly, data augmentation through supplementing GT training data with all eroded and dilated feature sets (see Fig 3). Models were evaluated at each level of erosion and dilation to assess the model’s robustness.

A second impact mitigation method was tested, using only a small subset of highly stable radiomic features to improve model robustness, balancing feature reliability and utility. Feature reliability was calculated via the intraclass correlation coefficient (ICC) of each feature across E × 1, D × 1, and GT feature sets. Feature utility was measured using average feature importances over the set of previously augmented models. Selected features required above average importance (≥0.053) and excellent reliability (ICC ≥ 0.9) [23].

## 3 Results

### 3.1 Exp. 1: Feature stability

The average absolute difference (relative to GT feature values) following subsequent iterations of erosion/dilation is reported in Table 3 for each of the 19 first-order radiomic features, alongside averages of these changes for each major diagnostic group (MB/PA/EP).

**Table 3:** Mean Absolute Deviation from GT Feature Values Following Erosion/Dilation.

Feature Name	E × 3	E × 2	E × 1	D × 1	D × 2	D × 3
10^th^ Percentile	0.11	0.09	0.06	0.17	0.35	0.49
90^th^ Percentile	0.27	0.21	0.17	0.47	0.83	1.02
Energy	0.36	0.26	0.15	0.20	0.40	0.59
Entropy	0.87	0.60	0.42	0.80	1.24	1.45
Interquartile Range	0.40	0.33	0.24	0.58	1.15	1.58
Kurtosis	0.62	0.55	0.46	0.68	0.81	0.92
Maximum	0.80	0.60	0.38	1.05	1.30	1.41
Mean	0.14	0.11	0.09	0.19	0.32	0.39
Mean Absolute Deviation	0.48	0.38	0.30	0.96	1.70	2.15
Median	0.11	0.08	0.06	0.09	0.16	0.22
Minimum	0.45	0.32	0.15	0.50	0.81	1.01
Range	0.93	0.69	0.41	1.18	1.56	1.75
Robust Mean Absolute Deviation	0.42	0.34	0.26	0.68	1.32	1.79
Root Mean Square	0.16	0.13	0.10	0.25	0.42	0.50
Skewness	0.60	0.46	0.37	0.66	0.73	0.76
Standard Deviation	0.53	0.43	0.33	1.19	1.98	2.41
Total Energy	0.40	0.30	0.17	0.23	0.46	0.67
Uniformity	1.11	0.64	0.43	0.56	0.85	0.99
Variance	0.36	0.30	0.23	1.16	2.22	2.87
Cohort Average	0.48	0.36	0.25	0.61	0.98	1.21
EP Average	0.61	0.47	0.34	0.73	1.09	1.25
MB Average	0.58	0.44	0.31	0.72	1.05	1.21
PA Average	0.60	0.46	0.34	0.90	1.53	1.96

*Features were z-score normalised prior to calculation using GT normalisation parameters.

Successive iterations of erosion and dilation demonstrate progressively stronger impact on feature values. However, the rate of this increase exhibited a diminishing trend, with the relative absolute difference displaying a wide variation between features. Dilation introduced considerably larger deviation for PAs, whereas EPs and MBs exhibited similar deviation to cohort average.

**Fig 4:**
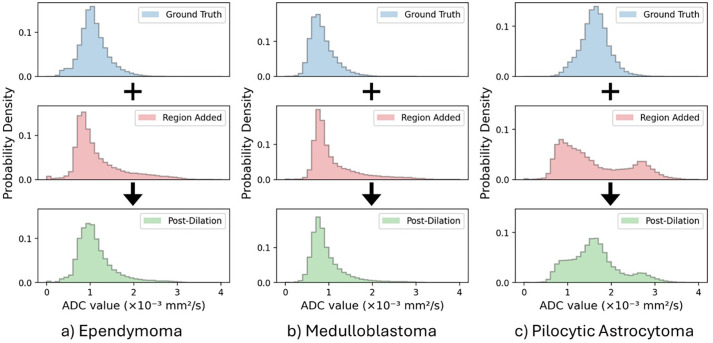
The resulting impact of 3 iterations of dilation on the average ADC intensity profile for a) Ependymomas. b) Medulloblastomas. c) Pilocytic Astrocytomas. The average ground-truth ADC profile is on top in blue, and the average dilated profile is on bottom in green. The average profile of the new region added through dilation (i.e., the difference between ground-truth and dilated ROIs) is in the middle in red.

The average ADC intensity profile of PAs is visibly impacted following ROI dilation. As shown in Fig 4, the region added by dilation for PAs exhibited a clear bimodal distribution of intensities, including clear peaks either side of ground-truth average ADC. Dilation demonstrated limited visual impact to average ADC intensity profiles for EPs and MBs.

**Fig 5:**
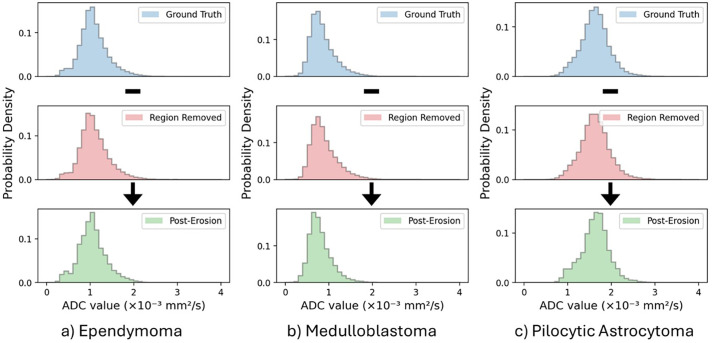
The resulting impact of 3 iterations of erosion on the average ADC intensity profile for a) Ependymomas. b) Medulloblastomas. c) Pilocytic Astrocytomas. The average ground-truth ADC profile is on top in blue, and the average eroded profile is on bottom in green. The average profile of the region subtracted through erosion (i.e., the difference between ground-truth and eroded ROIs) is in the middle in red.

As shown in Fig 5, erosion demonstrated limited visual impact on the average ADC intensity profile across diagnoses.

Table 4 contains results from the set of paired t-tests determining whether absolute differences relative to GT feature values are significantly greater following dilation (D × 1) or erosion (E × 1). Following a Bonferroni correction, 18/19 features exhibited a significantly (*p* < 0.0026) greater impact from dilation, with a large effect size for 11 features, a medium effect size for 3 features, and non-significant for Kurtosis only.

**Table 4:** Feature-wise Erosion (E × 1) vs Dilation (D × 1) paired t-Tests and Associated Effect Sizes.

Feature Name	t-statistic	p-value	Cohen’s d
Standard Deviation	15.63	<0.001	1.61
Variance	13.02	<0.001	1.49
Range	10.55	<0.001	1.41
Mean Absolute Deviation	13.81	<0.001	1.38
Maximum	8.51	<0.001	1.13
Entropy	14.13	<0.001	1.07
Robust Mean Absolute Deviation	10.49	<0.001	1.01
Root Mean Square	10.27	<0.001	0.96
Interquartile Range	9.67	<0.001	0.92
Mean	9.77	<0.001	0.86
90^th^ Percentile	8.00	<0.001	0.85
Minimum	6.20	<0.001	0.71
Skewness	4.77	<0.001	0.56
10^th^ Percentile	5.22	<0.001	0.55
Uniformity	6.42	<0.001	0.41
Energy	7.68	<0.001	0.36
Median	5.53	<0.001	0.36
Total Energy	6.38	<0.001	0.30
Kurtosis	2.31	0.023	0.28

*Two-tailed paired t-tests, αcorr.=0.0026.

### 3.2 Exp. 2: Optimal single ROI drawing strategy

**Fig 6:**
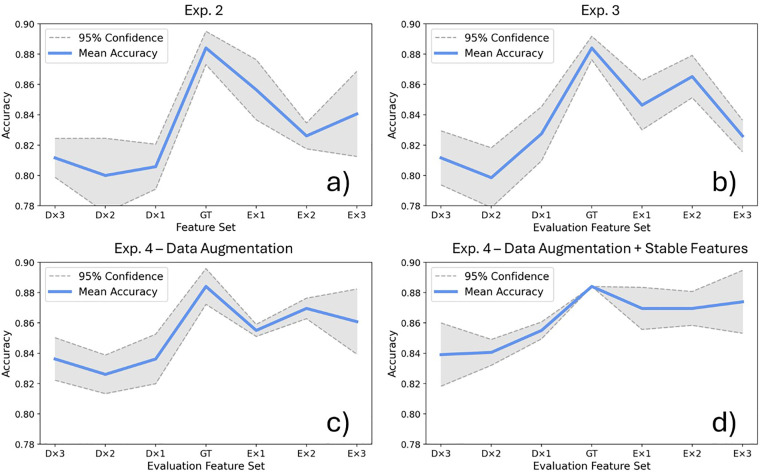
Average accuracy, shown in blue, of a) models when trained and evaluated using utilising ROIs at each level of erosion and dilation. b) models trained utilising only the GT ROIs, and evaluated on all levels of erosion and dilation. c) models trained and evaluated using ROIs at all levels of erosion and dilation. d) models trained and evaluated using ROIs at all levels of erosion and dilation, but with only the set of highly stable and useful features. The 95% confidence interval was calculated by repeating the experiment 100 times and is shown in grey.

As shown in Fig 6, models trained and evaluated with GT features performed better than models trained and evaluated with features extracted from all eroded or dilated ROIs. Erosion demonstrated a smaller impact on model performance than dilation, with single iterations of erosion (E × 1) and dilation (D × 1) reducing diagnostic accuracy by 2.7 ± 0.8% and 7.8 ± 1.0% respectively.

### 3.3 Exp. 3: Impact on existing trained diagnostic models

All models trained on GT features displayed small reductions in prediction accuracy when evaluated with non-GT features, with increased impact following subsequent iterations of boundary adjustment, as seen in Fig 6. Observed impact was larger under dilation than under erosion, with GT overall model accuracy reduced by 3.8 ± 0.8% and 5.6 ± 0.9% for E × 1 and D × 1 features respectively.

### 3.4 Exp. 4: Mitigating impact and improving model robustness

Models trained on the combination of all 7 feature sets (E × 1–3, D × 1–3, and GT) attained the highest performance when evaluated on GT features. As displayed in Fig 6, reductions in evaluation accuracy when using eroded and dilated feature sets was partially mitigated. Compared to GT evaluation accuracy, models exhibited average accuracy drops of 2.9 ± 0.2% and 4.8 ± 0.8% when evaluated on E × 1 and D × 1 features respectively.

Full average feature importances from these augmented models, and each feature’s ICC is available in Supplementary Material. The 6 features with above average feature importance in descending order were median, mean, 10^th^ percentile, Root-Mean-Square (RMS), 90^th^ percentile and minimum. The 9 features with ICC ≥ 0.9 in descending order were Energy, Median, Total Energy, Mean, 10^th^ percentile, RMS, 90^th^ percentile, Uniformity and Interquartile Range. Therefore, only Median, Mean, 10^th^ Percentile, RMS and 90^th^ Percentile were included in the reduced feature space.

Fig 6 illustrates that utilising reduced stable-feature sets and data augmentation yielded increased overall performance on the GT data, and improved robustness when evaluated on eroded and dilated features. Compared to GT evaluation performance, models demonstrated accuracy drops of 1.4 ± 0.7% and 2.9 ± 0.3% when evaluated on E × 1 and D × 1 features respectively. E × 2–3 had minimal further impact on evaluation accuracy compared to E × 1.

## 4 Discussion

### 4.1 ADC features & intensity profiles

Whilst all features exhibited increasing deviation from GT values following each iteration of erosion and dilation, dilation introduced significantly greater deviation than erosion. As visualised by Figs 4 and 5, this increased deviation arises from the inclusion of external non-tumorous tissues with markedly different ADC values, with PAs demonstrating the highest susceptibility. This contrasts with EPs and MBs, which exhibited ADC profiles more consistent with surrounding tissues, masking some impact of dilation.

PAs exhibit less restricted diffusion than other paediatric posterior fossa tumours such as MBs and EPs [8; 24], and increased presence of cystic regions both internally and externally. Therefore, PAs present a distinct, elevated ADC intensity profile, with minimal overlap with surrounding low-ADC white/grey matter and high-ADC cysts included through dilation, visibly altering post-dilation ADC intensity distributions.

Previous studies have focused on using cohorts comprised of individual diagnoses, using multiple segmentation perturbations to identify the most stable features within their specific domains [14–16; 25]. This work instead focused on comparisons between simulated biases, demonstrating dilation introduced greater feature value deviation than erosion across the diagnostically diverse cohort of paediatric brain tumours.

Further, whilst this work provides recommendations based on multiple diagnoses, the variability demonstrated between diagnoses and their susceptibility to ROI biases indicates individual diagnosis-specific ROI annotation guidance may be required.

### 4.2 Diagnostic model performance

Previous studies have investigated the impact of geometric manipulations such as rotation, translation and contour variation on the stability of many first-order radiomic features in a wide set of oncological domains [14; 15]. Such studies measure ICC to perform stable feature selection. This research additionally demonstrates that, simulating annotator volume biases impacts not only feature values but diagnostic utility.

Models trained and evaluated using ground-truth feature values outperformed those trained and evaluated using features from eroded or dilated ROIs, indicating that where possible, ROIs should be delineated as closely to boundaries as possible, rather than systematically promoting conservative or extensive drawing strategies. However, across all trained models, dilation had a significantly larger negative impact on model accuracy than erosion, indicating that where tumour boundaries are ambiguous, conservative approaches may yield quantitative features with greater diagnostic utility than approaches including ambiguous subregions.

### 4.3 Diagnostic impact mitigation

Following similar feature selection methodologies to the literature, balancing both feature stability and utility [25], combined with data augmentation, the impact of erosion is heavily mitigated. This provides further evidence that conservative annotation of ambiguous boundaries is preferable, resulting in minimal diagnostic impact. This supports Seetha, et al. [26], who demonstrated that by utilising a set of stable and relevant radiomic features, the impact of ROI variation can be partially mitigated.

Mean and median ADC values were identified as the most diagnostically important features. These highly correlated values, have consistently demonstrated good diagnostic value [7; 16; 24; 27; 28], and have been demonstrated in this study to also be robust against significant annotation bias.

### 4.4 Limitations

Previous studies have placed significant emphasis on individual feature stability, and care should be taken in attributing instability of some features to the specific domain, given their instability in uniform phantom studies where the only changing factor is ROI volume [29], implying a level of inherent mathematical instability of some features. However, in practice this research has demonstrated the benefits of robust feature selection and its ability to partially mitigate instability stemming from biological heterogeneity.

Findings are constrained to diagnostic model performance and require generalisation to other downstream tasks such as prognostics and treatment planning. Whilst dilating ground-truth annotations does not improve diagnostic performance, activity external to tumours such as tumour infiltration, oedema and cysts may be captured by extensive boundaries and provide meaningful insight to other downstream tasks. Further work should extend this study to prognostic domains prior to clinical translation of quantitative prognostic models.

Furthermore, due to an insufficient number of training cases, the diagnostic task was limited to only three diagnostic groups covering 6/25 distinct diagnoses in this study’s cohort. Therefore, these trends in diagnostic performance, whilst helpful, do not fully capture the full diagnostic landscape of paediatric brain tumours, excluding 54% of clinical cases [17].

Finally, the simulation of alternative ROI drawing methodologies does not perfectly capture real annotator biases, with strict adherence to ground-truth geometry rather than imaging contours. Dilated ROIs included some evidently erroneous regions such as cysts or CSF. Whilst post-processing steps such as thresholding to exclude such hyper/hypointense regions may have mitigated this impact, exact threshold values and other post-processing parameters are subjective with significant variability observed between the average ADC of cystic regions [30; 31]. Instead, human annotators should be utilised in future work to validate these findings and assess the impact of different drawing strategies and guidance on radiological diagnostic tools under clinical constraints.

### 4.5 Conclusion

Accurately drawn ROIs demonstrate superior utility for quantitatively diagnosing paediatric brain tumours, with the simulated conservative and extensive ROIs negatively impacting diagnostic accuracy. The smaller impact from using conservative boundaries over extensive boundaries, indicates that the exclusion of ambiguous boundary regions is preferable over their inclusion for diagnostic tasks employing first-order ADC radiomics. The augmentation of training data using the simulated conservative and extensive tumour boundaries, alongside stable-feature reduction, demonstrated improved model robustness, reducing the overall performance impact of sub-optimal drawing strategies.

## Supporting information

S1 File Supplementary Material. Additional data concerning full diagnostic labels and feature importance for radiomic features.
(DOCX)
