# Supplementary material for: Evaluating the impact of segmentation strategies on radiomic feature stability for paediatric brain tumour diagnosis using diffusion weighted imaging
Source: PLoS One. 2026 Aug 18;21(8):e0356382. doi: 10.1371/journal.pone.0356382 (PMC13484970; doi:10.1371/journal.pone.0356382)
Supplement: Supplementary file 1 [file pone.0356382.s001.docx]

# Supplementary Material

Table A: Full Cohort Diagnosis Incidence

| **Diagnosis** | **Number of Cases** |
| --- | --- |
| Pilocytic Astrocytoma | 30 |
| Medulloblastoma | 23 |
| Glioblastoma | 8 |
| Anaplastic Ependymoma | 6 |
| Ependymoma | 6 |
| Unbiopsied Optic Pathway Glioma | 4 |
| Desmoplastic-Nodular Medulloblastoma | 3 |
| Ganglioglioma | 3 |
| Germinoma | 3 |
| Anaplastic Astrocytoma | 2 |
| Choroid Plexus Carcinoma | 2 |
| Pilomyxoid Astrocytoma | 2 |
| Subependymal Giant Cell Astrocytoma | 2 |
| Anaplastic Medulloblastoma | 1 |
| Astroblastoma | 1 |
| Atypical Choroid Plexus Papilloma | 1 |
| Atypical Teratoid - Rhabdoid Tumour | 1 |
| Choroid Plexus Papilloma | 1 |
| CNS Primitive Neuroectodermal Tumour | 1 |
| Dysembryoplastic Neuroepithelial Tumour | 1 |
| Mixed Germ Cell Tumours | 1 |
| Pineoblastoma | 1 |
| Teratoma - Immature | 1 |
| Teratoma - Mature | 1 |
| Unbiopsied Secreting Germ Cell Tumour | 1 |

Table B: Average Feature Importance For Augmented Diagnostic Model and Feature Stability

| **Feature Name** | **Feature Importance** | **ICC** |
| --- | --- | --- |
| 10^th^ Percentile | 0.170 | 0.98 |
| 90^th^ Percentile | 0.079 | 0.91 |
| Energy | 0.007 | 0.99 |
| Entropy | 0.009 | 0.87 |
| Interquartile Range | 0.009 | 0.90 |
| Kurtosis | 0.011 | 0.61 |
| Mean Abs. Difference (MAD) | 0.005 | 0.84 |
| Maximum | 0.004 | 0.77 |
| Mean | 0.206 | 0.98 |
| Median | 0.228 | 0.99 |
| Minimum | 0.060 | 0.81 |
| Range | 0.006 | 0.81 |
| Root Mean Square | 0.145 | 0.97 |
| Robust MAD | 0.007 | 0.87 |
| Standard Deviation | 0.004 | 0.82 |
| Skew | 0.025 | 0.76 |
| Total Energy | 0.014 | 0.99 |
| Uniformity | 0.008 | 0.91 |
| Variance | 0.004 | 0.84 |

*ICC was calculated between E×1, D×1, and GT Features.
